# Supplementary material for: Machine learning models to predict traumatic brain injury outcomes in Tanzania: Using delays to emergency care as predictors
Source: PLOS Glob Public Health. 2023 Oct 19;3(10):e0002156. doi: 10.1371/journal.pgph.0002156 (PMC10586611; doi:10.1371/journal.pgph.0002156)
Supplement: Attachment — Submitted filename: Response to reviewers.docx [file pgph.0002156.s001.docx]

Thank you very much for taking the time to review our manuscript. Your feedback is very much appreciated. Please find our responses to reviewer and editor comments below. We hope we have addressed your concerns.

**Reviewer 1 comments**

**Comment**

The authors are interested in creating a tool for prognostication for traumatic brain injury (TBI) in low and middle income countries (LMIC’s). They build upon previous TBI prognostication models by adding elements of delay to care. They use data from TBI patients admitted to a single institution in Kenya to derive this model. I thank the authors for addressing a hugely important issue in trauma and global health. TBI is a massive problem worldwide, but especially in LMICs. Any practical tools that can be developed and deployed are helpful. Generally, the concept and methodology for this manuscript are sound. I have only a few comments:

**Response**

Thank you very much for this comment.

**Comment**

Introduction: The authors do a good job of explaining the 3 delays. In the description of the reasons for the first delay they ascribe it primarily to an individual’s perceived need. I would add that a big part of it is not just the individuals perceived need but also their perceived ability to reach care that is available, effective and affordable. It is common for an individual to know that they need care but may delay in seeking care because of these other barriers.

**Response**

Thank you for this comment. We completely agree and now state the following in the introduction of our revised manuscript: “Delays in seeking care are largely dependent on an individual’s perceived need for care and their ability to access effective and affordable treatment, both of which can be influenced by cultural, geographical, and socioeconomic factors.”

**Comment**

Introduction: The authors state that healthcare providers can only influence the third delay. This statement ignores the power of research, policy and advocacy work by healthcare providers that is critical for effecting change on the first two delays.

**Response**

Thank you for this comment. To capture the substantial role that research, policy, and advocacy play in reducing delays to care we have removed the following sentence from the introduction section of our revised manuscript “Of all three delays, ED healthcare providers can only influence time to treatment after a patient reaches the hospital” and replaced it with “ED healthcare providers can play a critical role in reducing all three delays through research, policy, and advocacy.”

**Comment**
Methods: Line 134: “which” -> “whom”

**Response**

Thank you for this comment. We have made this grammatical change in the revised manuscript.

**Comment**
Results: Line 244: this is a minor point but it isn’t a “majority” if it is less than 50%

**Response**

Thank you for this comment. We have revised the sentence in question to the following: “In both the good and poor outcome groups the *most common* time to ED arrival was more than 12.0 hours after injury occurrence (31.6%, 33.3%).”

**Comment**
Discussion: My main concern with the paper is operationalization of the model. 12 separate time variables seems like a lot of data to be able to collect and use. Now, the authors created multiple models and some of these might have used only some of the variables. But it is difficult to know, from the way the manuscript is currently written, what goes into each model. The details about which variables ended up in each model would be helpful to include in the results and in a figure.

**Response**

Thank you for this comment. Each model in this study used the same set of predictor variables. To make this clear to the reader, we now state the following under the “Study Variables” heading of the results section of our revised manuscript: “Each of the models assessed in this study included the following variables as predictors: age, sex, mechanism of injury, intention of injury, day of injury alcohol use, temperature, respiratory rate, heart rate, systolic and diastolic blood pressure, pulse oxygen, pupil reactivity, Glasgow Coma Score (GCS), ED disposition, and twelve time to care variables. The twelve time to care variables included…” In addition, to make clear to the readers how data for the time to care variables was collected we also state the following in the revised manuscript “Time stamps for each event were recorded and entered into the TBI registry upon patient encounter/event occurrence. Our time to care variables were then calculated in the background of our registry as the difference between time stamps for different events.” In the discussion section of our revised manuscript we also now provide a commentary on the practicality of collecting time stamp data in resource limited settings.

**Comment**

Discussion: For particle purposes there is always a tradeoff between the model with the best area under the curve (AUC) and the one that is most user friendly. The authors make some effort to address this in the section on “Usability and Time to Care.” I think the authors could be more specific about which of the 12 variables they recommend. I also think that they are overly optimistic about how easy it will be to collect time stamps on all of these events. Time stamps of events in trauma care are difficult in HICs and can be extremely difficult in settings where resources and record keeping are more difficult.

**Response**

Thank you for this comment. Under the “Usability and Time to Care” heading of the discussion section in our revised manuscript, we have toned down our optimism regarding the ease of collecting trauma time stamps and now show regard for the difficulty of collecting such data in resources limited settings. In addition, in this section we also now suggest, based on figure 2, which time to care variables should be prioritized if data collection efforts are limited. Specifically, we now say: “In addition, if collecting information on all twelve time to care variables proves to be cumbersome, users could also prioritize those variables which we show to be most important to model prediction (ex. time to brain CT scan, time to oxygen, time to ICU admission, and time to fluids).” Lastly, as noted in our response below, while we want to focus this paper on the addition of time to care variables to our baseline models, more work is needed to elucidate whether or not there is a subset of time to care and or other variables that achieves the same level of prediction as the models presented in this study. We are planning to conduct such an analysis in a future study and provide further details under the “Future Steps” heading of our discussion section in our revised manuscript.

**Comment**

Discussion: The authors present their models but then stop short of making a recommendation. I am most interested to hear which variables are most important for prediction. Therefore, figure 2 is helpful. I am also interested in how good the model is at prediction. Therefore, figure 1 is helpful. But it would be most useful to know the details on the models so these data can be operationalized. It would also be helpful to know if a very simple model (for instance, one that just includes the time variables on time to CT scan, GCS and O2 sat) is almost as good as a model.

**Response**

Thank you for this comment. As noted above, to ensure readers understand which variables are included in each model we now state the following under the “Study Variables” heading of the results section of our revised manuscript: “Each of the models assessed in this study included the following variables as predictors: age, sex, mechanism of injury, intention of injury, day of injury alcohol use, temperature, respiratory rate, heart rate, systolic and diastolic blood pressure, pulse oxygen, pupil reactivity, Glasgow Coma Score (GCS), ED disposition, and twelve time to care variables. The twelve time to care variables included…” In addition, we completely agree that it is important to know if a very simple model with few variables would be just as good as the models presented in this paper. However, we wanted the focus of this paper to be on whether the addition of time to care variables improves performance of our baseline models, and thus whether our time to care variables may augment TBI prognostication. We are planning to use a feature selection approach in a future study, with more/updated data from our registry, to elucidate the most parsimonious model. To make this clear to the reader, we now state the following under the “Future Steps” heading of the discussion section of our revised manuscript: “It is also important to note that the most practical prediction model is one that achieves a desired level of performance with the fewest number of predictors. In a future study, with updated data from our TBI registry, we plan to use a feature selection approach to identify whether or not there is a subset of our time to care and or clinical/sociodemographic variables that produces a more parsimonious model, and therefore a more practical model for implementation in resource limited settings.”

**Reviewer 2 comments**

**Comment**

In their study the authors present a TBI outcome predictive algorithm based on machine learning capabilities. Although registry-based data often lack granularity, this limitation seems to have been somehow addressed by the authors, as some supplementary data was extracted in an attempt to evaluate the need for different procedures. This is especially relevant in cases of LMICs where inaccessible or costly yet necessary procedures may be omitted which in turns may influence the certainty in the diagnosis, the treatment of choice, patient outcomes, and in that context, the data on hand. Adding information about the unmet needs for a procedures adds to the credibility of the dataset, which the authors ought to be commended for. The results seem to provide evidence backing up the role of delay parameters in the prediction of outcomes after TBI injuries in lower resource settings. These findings are valuable, especially in the era of growing global health efforts, and may be used to guide future research as well as financial efforts and investments aiming at reducing mortality and morbidity from TBI.
However, some concerns are raised as following:

**Response**

Thank you for this comment.

**Comment**

Methods should better describe how TBI was defined? Were patients with severe injury to the skull also considered, where those with so-called “mild-TBI” included? This is essential to assess the external validity of the findings.

**Response**

Thank you for this comment. Our registry included patients with acute TBI (defined as TBI less than 24 hours since injury occurrence) of any severity. To make this clear we now state the following in the methods section of our revised manuscript under the “Study Participants” heading: “Patients were included in the registry if they presented with acute TBI (less than 24 hours since injury occurrence) of any severity and were evaluated by a physician in the ED. TBI patients who did not survive long enough to be evaluated by a physician in the ED, who presented for follow-up, or who presented with non-acute TBI were not included in the registry.”

**Comment**

Did the authors reflect over the possibility of a selection bias since the registry only includes patients presenting within 24-hours from their injury. This would not have been a problem in other study, however, in lower income settings (where delayed presentation is particularly common), and especially when the main aim of the study is to look at delays, such restrictions to the selection of patients warrants careful considerations, as they may limit generalizability of the findings in LMICs. This should hence at least be mentioned in the limitation section of the manuscript.

**Response**

Thank you for this comment. To ensure readers understand the selection bias present in our study, we now state the following in the limitations paragraph of our discussion section in our revised manuscript: “Second, the registry used in this study only includes patients who sustained a TBI no more than 24 hours prior to hospital arrival. Consequently, the registry likely underrepresents the most severe cases of TBI (i.e. those who died of their injury before reaching the hospital). Given that these patients are expected to benefit the most from timely care, the selection bias inherent in our registry likely underestimates the predicative power of our time to care variables.”

**Comment**

Percentages in Tables 1 and 2 and those described in the text look at the same thing from two totally differing angles which makes it harder to follow what is being referred to. For example, take the time to ED arrival variable. In the text the percentages seem to be expressed as the number of those presenting to the ED at more than 12.0 hours in relation to the total number of patients with either good or poor outcomes – which I personally prefer. The authors mention: “A majority of patients in both the good and poor outcome groups arrived to the ED more than 12.0 hours after injury occurrence (31.6%, 33.3%)” (page 8 line 244). However, in the table the authors calculate the percentages of patients with good/bad outcomes in relation to the total number of patients presenting at more than 12.0 hours (88.2% / 11.8%). I think the author should stick with one way of presenting findings, to avoid conclusion. I would recommend sticking with the percentages used in the text, meaning that the ones in the table may be changed.

**Response**

Thank you for this comment. We appreciate that it may be confusing to the reader to present our percentages from two different angles. Therefore, as suggested, in our revised manuscript we have changed the percentages in Tables 1 and 2 to match the percentages described in the text.

**Comment**

In addition, it is essential mentioning that although the delay-parameters seemed to improve the accuracy of the predictive model, the AUC ultimately experienced an absolute increase of 5% at best (range: 0.8-5%). I think the authors should spend a bit more time discussing and elaborating on whether the magnitude of this change is in fact clinically relevant and significant. Because, at a first glance, a maximal increase of 5% in accuracy only, may reflect a weak weighing coefficient associated with delay factors, although Figure 2 shows otherwise, especially to unexperienced readers. The authors should hence strive to better explain the relatively low improvements in accuracy after adding delay factors and how such result are able to translate to the findings presented by Figure 2 (or in other words, to the fact that delay factors held such a large share of the contribution in the ML predictive model).

**Response**

Thank you for this comment. We understand that a 5% increase in AUC and other performance metrics may or may not be clinically significant. We have therefore added the following text to the limitations section of the discussion in our revised manuscript: “Lastly, it is important to highlight that for most models the addition of time to care variables increased model performance metrics by no more than five percentage points. While such an increase indicates the predicative power of time to care variables, the clinical significance of a five-percentage point increase in AUC and other performance metrics can only be assessed through additional studies that measure the costs and benefits of using our models in clinical practice to facilitate TBI prognosis.”

**Comment**

In the paragraph usefulness, the authors highlight how their ML-based model can be used out in clinical practice. The authors also give examples as to how that may be the case. However, this whole section depends on the premise of the availability of their ML-algorithm. Is the algorithm available to the public, is there a publicly available app or a domain where it may be tested? If not, the authors may benefit from toning down on the usefulness aspect of their discussion and refrain from alluding to the practicality of their algorithm as a tool to use in the clinics. Consider hence removing the parts from line 348 to 357 of page 13, also since the examples provided are very impractical in a real-life situation. Instead, authors should focus on the valuable insight provided by their study (the fact that delays do play a role in outcomes) and suggest that this information may be taken into account when building actual tools for the early detection of unfavorable outcomes in TBI patients.

**Response**

Thank you for this comment. Our ML-based algorithms are not publicly available, nor have they been used/tested in actual clinics. However, despite this, we feel that it is still of merit to discuss the theoretical relevance/practicalities of using our algorithm in a clinical setting. That being said, we have toned down the text as suggested and have added additional text to this paragraph to highlight the importance of considering delays to care when building tools for TBI prognosis.

**Comment**

At the end of their discussion the authors mention: ”We plan to quantify associations between in-hospital emergency care delays and TBI outcomes in a future study”. Could you please explain how such a study would be different from this one? In this study the authors looked at both out- as well as in-hopital delays also, isn’t that right?

**Response**

Thank you for this comment. We refer to a study in which we would quantify the strength of correlation between care delays and different TBI outcomes using other statistical methods. However, we understand that simply saying this may be confusing to the reader (and not of much worth) so we have deleted this sentence altogether.

**Comment**

In the conclusion the authors mention that the predictors were ”novel”. From my understanding of the author’s introduction and methods section, these variables were had previously been described. I hence recommend removing the word ”novel”.

**Response**

Thank you for this comment. We have removed the word “novel” as suggested.

**Editor comments**

**Comment**

The addition of these delay variables is not trivial in the real world (per R1) and the final models only have marginal gains in AUC (per R2). This needs more discussion on the true utility of what you are proposing.

**Response**

Thank you for this comment. In the discussion section of our revised manuscript, we elaborate on the utility of our models. More specifically, we convey that the main utility is improved TBI prognostication and we highlight the importance of considering delays to emergency care in future TBI tools. In addition, we speculate on other theoretical advantages of our model including the ability of our time to care variables to inform the provision of specific clinical procedures and the usefulness of our time to care variables in settings where resources may prevent the collection of other more clinically complex indicators that predict TBI outcomes. That be being said, we understand that the marginal gains in AUC bring into question whether these gains are clinically significant. To address this, we have added the following text to the limitations section of our revised manuscript: “Lastly, it is important to highlight that for most models the addition of time to care variables increased model performance metrics by no more than five percentage points. While such an increase indicates the predictive power of time to care variables, the clinical significance of a five-percentage point increase in AUC and other performance metrics can only be assessed through additional studies that measure the costs and benefits of using our models in clinical practice to facilitate TBI prognosis.”

**Comment**
Many of the most predictive delay variables (per Figure 2) are that items were "not needed" (Brain Scan, Time to Oxygen, Fluids, ICU) or are items Needed and not received (Brain Scan, Oxygen). For the first list, these all have much lower rates of worse outcomes - this isn't a "time to get" but rather a "good enough they don't need". So is this really a delay variable, or some proxy for wellness? For the latter list, is it possible that these are needed but not received because the negative outcome already happened - for example, very sick, we couldn't get the brain scan? So again, this isn't really a delay variable but rather proxy for really sick? Very few of the "important delay predictors" are truly measures of time, but rather measures of not received with needed versus not, which I suspect makes these just proxy measures for very sick versus not. This needs more discussion, particularly on how these get captured and used in both model development and prediction deployment.

**Response**

Thank you for this comment. It is correct that the categories “not needed” and “needed, but not received” are ultimately proxies for wellness/sickness. However, we felt it important to include these categories in our time to care variables for practicality, because delays to care may not be relevant to every TBI patient. For example, mild TBI patients who do not require fluids will not be administered fluids and therefore will not experience delays in receiving fluids. The text under the heading “Additional Categories for Time to Care Variables” in the methods section of our revised manuscript describes how the categories “not needed” and “needed, but not received” were captured for use in model development. In addition, we have added additional text to the paragraph under the heading “Clinical Relevance and Time to Care” in the discussion section of our revised manuscript to elaborate on how these categories might be used in clinical practice/prediction deployment.

**Comment**

"Prognostic models are an innovative solution that can help optimize clinical decision making to ensure patients with the greatest need and potential benefit receive care first." This is an interesting framing, given that getting care first could mean delays for others. And delays captured in your model could be results of non-algorithm driven risk assessments. I have a hard time wrapping my head around this chicken-and-egg reasoning, and so ask that authors to add more here.

**Response**

Thank you for this comment. To avoid any confusion, we have altered this sentence to the following in our revised manuscript: “Prognostic models are an innovative solution that can help optimize clinical decision making to maximize positive patient outcomes.”

**Comment**

The authors report percent accuracy, sensitivity, etc, but never talk about how they decided what algorithm thresholds for classification for which these measures are reported.

**Response**

Thank you for this comment. In our revised manuscript we now state the decision threshold used to compute accuracy, sensitivity, specificity, PPV, and NPV. More specifically, under the heading “Predicative Modeling” in the methods section we state: “However, we also report accuracy, sensitivity, specificity, positive predictive value (PPV), and negative predictive value (NPV) using a standard classification threshold of 0.50 for all models.”
